# To the Editor Re: Miller et al., Occupational Protection in Interventional Radiology. A Joint Guideline of the Cardiovascular and Interventional Radiological Society of Europe and the Society of Interventional Radiology: a comment on occupational protection guidelines and the potential role of antioxidants

**DOI:** 10.1186/s42155-026-00709-4

**Published:** 2026-06-15

**Authors:** James A. Kelly, Gaylene Pron, Kieran Murphy

**Affiliations:** 1 Department of Vascular Surgery, University of Toronto, University Health Network, Toronto, Canada; 2 Institute of Health Policy, Management and Evaluation, Dalla Lana School of Public Health, University of Toronto, Toronto, ON Canada; 3 Department of Medical Imaging, Division of Neuroradiology, University of Toronto, University Health Network, Toronto, Canada

Dear Sir/Madame,

We read with disappointment the recent joint guideline from *the Cardiovascular and Interventional Radiological Society of Europe (CIRSE)* and *the Society of Interventional Radiology (SIR)* on occupational protection in interventional radiology, which provides a historically accurate framework for minimizing occupational radiation exposure through physical and procedural safety measures [1]. The Societies’ Writing Panel advocated for meticulous shielding, dose monitoring, and ergonomic optimization. Practice guidelines on radiation safety, have also been made recently by the *European Society for Vascular Surgery (ESVS)* [2]. Recommendations by these Societies are fundamental to contemporary interventional practice and remain at the forefront in advocating for safety of all interventionalists, as they have for the past 40 years. However, the *ESVS* additionally took a forward-looking review for their guidelines on new and future technologies impacting radiation and interventional practice such as three-dimensional navigation, robotic tracking, and artificial intelligence. They also included a significant section on patient and public perception of radiation safety and their informational needs regarding radiation risks during procedures.

We noted that the Writing Panel of the *CIRSE/SIR* guideline also reported that “certain parts of organs and tissues (brain, lateral chest wall, and axillary portions of the breast) are not fully shielded” from scatter radiation with current personal protective equipment or room structural modifications. Therefore, the need for additional protection to ionizing radiation would likely be clinically important. It may also be important that some individuals may need more protection than others against radiation-induced adverse effects because of their sensitivity to DNA damage or their inability to repair DNA damage from occupational radiation exposures [3–5]. Based on the variability in biological responses of individuals to occupational radiation exposure, some have suggested that safety levels based only on physical dosimetry do not accurately account for their radiation risks [6, 7]. The variability between individuals in their biological responses to radiation induced DNA damage were also discussed in the *ESVS* guideline report and suggestions were made that genetic profiling may identify those at greater risk from their occupational radiation exposure.

We wish to express an underrepresented dimension of occupational radiation protection in the *CIRSE/SIR* guideline report namely; the potential role of antioxidant-based interventions in mitigating radiation-induced cellular injury. Ionizing radiation exerts its biological effects largely via generation of reactive oxygen species (ROS), which in turn results in oxidative stresses leading to DNA damage and activation of inflammatory signaling pathways [8]. This provides a biologically plausible rationale for exploring antioxidant supplementation as a strategy to complement standard safety care to limit radiation-induced subcellular damage.

Although a few references were cited on the role of antioxidants by the *CIRSE/SIR* Writing Panel, the evidence search strategies were not reported in the Methods and any discussion and summary judgements on this topic was likely beyond the scope of this guideline report. However, several experimental and early human studies on antioxidants have demonstrated encouraging findings.

One such paper—briefly mentioned in the Guideline—is a pilot study of patients undergoing cardiac catheterization who were prescribed oral ascorbic acid prior to radiation exposure versus controls [9]. Patients taking ascorbic acid were associated with a higher reduced/oxidized glutathione ratio than controls. Similarly, a randomized pilot study in patients undergoing diagnostic radiation exposure demonstrated that a multi-ingredient antioxidant regimen significantly attenuated radiation-induced DNA double-strand breaks measured by γ-H2AX [10]. In preclinical models, combined antioxidant formulations have been shown to reduce oxidative stresses, inflammatory pathway activation, and mitochondrial DNA damage following ionizing radiation exposure [11]. Furthermore, observational data in healthcare workers suggest that higher dietary antioxidant intake is associated with improved redox balance in individuals chronically exposed to low-dose radiation [12]. Finally, a recent systematic review of antioxidant use during radiotherapy, whilst admittedly not focusing on occupational exposure, noted antioxidants reduced patient side effects of mucositis, dermatitis, and xerostomia and could highlight the wider potential benefits also among interventionalists alike regularly encountering radiation exposure [13].

These findings suggest a biological signal that antioxidant-based interventions may modulate early radiation-induced cellular injury pathways. This evidence, spanning mechanistic, pre-clinical, and small clinical studies provides a translational continuum that warrants further investigation, and we argue, a greater consideration in future society guidelines and recommendations.

We acknowledge that the current evidence base is usually limited by small sample sizes, short follow-up, heterogeneity of the antioxidant formulations, and reliance on surrogate biomarkers rather than clinical endpoints such as cancer incidence. We note that authors of the *ESVS* practice guidelines state that many of their recommendations, other than those involving physics principles, were based on Level C evidence and reliant on expert opinion of committee members. The absence of robust long-term randomized trials should therefore not preclude consideration of biologically plausible adjunctive strategies in occupational radiation safety research.

Given the chronic cumulative exposure experienced by interventional radiology personnel, further prospective and randomized studies evaluating well-characterized antioxidant regimens in occupational cohorts may be valuable in determining whether modulation of oxidative stress pathways can complement established physical radioprotection measures. Discussion through such society guidelines is often what initiates and drives recruitment of such future trials; findings which could have beneficial impacts upon us all and our future trainees. We therefore suggest that future iterations of occupational radiation safety guidelines may benefit from conducting more comprehensive evidence reviews on antioxidants and acknowledging biological radioprotective strategies as an emerging area of investigation alongside traditional engineering and procedural controls.

We wish to end by thanking all participants and society members for their efforts in the production of these guidelines.

## Data Availability

Publicly available.
